# Present, old and future strategies for anti-HCV treatment in patients infected by genotype-1: estimation of the drug costs in the Calabria Region in the era of the directly acting antivirals

**DOI:** 10.1186/1471-2334-14-S5-S3

**Published:** 2014-09-05

**Authors:** Alessio Strazzulla, Chiara Costa, Vincenzo Pisani, Vincenzo De Maria, Francesca Giancotti, Sebastiano Di Salvo, Saverio Giuseppe Parisi, Monica Basso, Marzia Maria Franzetti, Nadia Marascio, Maria Carla Liberto, Giorgio Settimo Barreca, Angelo Giuseppe Lamberti, Emilia Zicca, Maria Concetta Postorino, Giovanni Matera, Alfredo Focà, Carlo Torti

**Affiliations:** 1 Unit of Infectious Diseases, University "Magna Graecia", Catanzaro, Italy; 2 Unit of Hepatology, Hospital "Mater Domini", Catanzaro, Italy; 3 Department of Molecular Medicine, University of Padua, Padua; 4 Institute of Microbiology, Department of Health Sciences, "Magna Graecia" University of Catanzaro; 5 University Unit of Infectious Diseases, University of Brescia, School of Medicine, Brescia, Italy

**Keywords:** Cost, SVR, DAA, HCV, Telaprevir

## Abstract

**Background:**

In Italy, anti-HCV drugs are provided free of charge by the National Health System. Since 2011, three drug regimens including a directly acting antiviral (DAA) are considered the gold standard for HCV treatment. However, these drugs add a significant cost (roughly €26,000) to the combination of pegylated-interferon-α/ribavirin (PEG-IFN/RBV), which before DAA represented the unique treatment. To provide the National Health System potential useful information, we estimated costs to provide anti-HCV drugs to treat a population experienced for PEG-INF/RBV.

**Methods:**

Genotype 1 HCV mono-infected or HIV/HCV co-infected individuals who were treated with PEG-IFN/RBV between 2008 and 2013 were included. The cost to treat these patients with PEG-IFN/RBV was calculated (cost 1). We also estimated costs if we had to treat these patients with a lead-in period of PEG-INF/RBV followed by PEG-IFN/RBV and a DAA in naïves (cost 2), in addition to cost 1 *plus *the estimated cost to re-treat with PEG-IFN/RBV and a DAA patients who had a relapse or a non response (cost 3). Moreover, all costs were normalized by SVR. Rates of foreseen response with DAA were obtained from literature data.

**Results:**

The overall study population consisted of 104 patients. The rate of sustained virological response (SVR) was 55%, while it was estimated that SVR would be obtained in 75% of patients with a lead-in period with PEG-IFN/RBV followed by a DAA combination, and in 78% if this treatment is used to re-treat experienced patients with a DAA. Drug costs associated with these treatments were: €1,214,283 for cost 1, €3,474,977 for cost 2 and €3,002,095 for cost 3. Costs per SVR achieved were: €22,284 for cost 1, €44,643 for cost 2 and €38,322 for cost 3.

**Conclusions:**

Treatments including DAAs achieve a SVR in more patients than PEG-IFN/RBV but they cost around three times more than PEG-IFN/RBV alone regimens. Also, cost per SVR is almost twofold greater than PEG-IFN/RBV regimens. Therefore, it is mandatory to implement use of DAA in clinical practice, but the National Health System should allocate adequate resources to provide drugs, which challenges sustainability. Cost reduction for anti-HCV drugs should be pursued.

## Background

It is estimated that hepatitis C virus (HCV) infects more than 170 million people worldwide, with 17 million of them living in the Mediterranean region. In Italy, prevalence is 3-4.4%, with peaks reported in Southern regions (12.6-26%) [1-4]. The standard of care for treatment has been represented by a combination of pegylated interferon-α (PEG-IFN) and ribavirin (RBV) for all HCV genotypes. However, the treatment response of genotype 1 HCV after PEG-IFN/RBV is not acceptable when compared to PEG-IFN/RBV in combination with the new directly acting antivirals (DAA), which are now recommended as the standard of care for treatment of patients infected by this genotype [5,6]. Currently, in Italy, boceprevir (BOC) and telaprevir (TPR) are the only available and recommended DAAs [7].

In Italy, current recommended drugs are provided free of charge to patients in need. DAA's increase the cost of therapy to a significant extent (by roughly €26,000). For this reason, in the current economical crisis and from the payer perspective of National Health System (NHS), it is difficult to convince authorities to extend prescription of these regimens to a large number of patients. In addition, it is important to provide authorities with estimates of resources needed to treat patients.

With this objective in mind, we calculated the actual costs to treat these patients and estimated costs to treat with a DAA the same population of naïve patients or to re-treat with a DAA only those who did not respond to the previous regimen (PEG-IFN/RBV).

## Methods

The study was conducted in three Units, two located in Southern Italy (Hepatology Unit of the University Hospital *"Mater Domini" *Catanzaro and Infectious Disease Unit in the same Hospital), and one located in Northern Italy (Unit of Infectious Diseases of the University of Padua). Patients from the University of Padua were co-infected with human immunodeficiency virus (HIV), while those from the University Hospital *"Mater Domini" *in Catanzaro were HCV mono-infected. The study was conducted under the provisions of the Declaration of Helsinki, and in accordance with the International Conference on Harmonization Consolidated Guideline on Good Clinical Practice. As this study was retrospective and non-pharmacological, written informed consent has not been provided. Approval was obtained from the local ethical committee of the "Mater Domini" Teaching Hospital.

An observational study was performed, including all HCV-genotype 1 infected patients, treated with PEG-IFN/RBV from January 1, 2008 to June 30, 2013. A cost-consequences analysis was assessed and incremental costs of therapies were computed and listed.

We compared the actual cost for treatment of naive patients with PEG-IFN/RBV (cost 1), versus the estimated cost that would have been spent to treat the same patients with PEG-IFN/RBV in combination with a DAA (cost 2), versus cost 1 *plus *the estimated additional cost for retreatment of patients who did not respond to PEG-IFN/RBV if a DAA would have been added for re-treatment (cost 3).

For cost 2 estimation, initial treatment for 4 weeks (so called "lead-in") with PEG-IFN/RBV was assumed prior to the addition of a DAA for 12 weeks (if needed depending on achievement of a rapid virological response, RVR), then followed by PEG-IFN/RBV for 12 to 36 weeks depending on HCV RNA levels at weeks 4 and 12 [7,8]. Costs were normalized for sustained virological response (SVR) derived from results of the ADVANCE trial [9]. According to this trial, a 75% rate of SVR in patients naïve to PEG-IFN/RBV was used for estimating cost 2.

For cost 3, the estimated cost for retreating with PEG-IFN/RBV and a DAA (in case of a non response or relapse) was added to cost 1. We hypothesized that 12 weeks of a 3-drug regimen is prescribed, followed by 36 weeks of PEG-IFN/RBV combination in all patients [7]. Proportions of SVR for cost normalization (cost per SVR achieved) were derived from the REALIZE trial [10]. They were 29% for null responders, 59% for partial responders and 83% for patients who had a virological relapse.

Drug costs per week were based on an article by *Cammà et al*. [11]: €165.57 for PEG-IFN, €106.15 for RBV and €2,083.00 for TPR (assumed as reference DAA). Discounted rate was not applied for cost estimations.

## Results

A total of 104 patients were included in the study. Patient characteristics and response to treatment (either actually achieved or estimated for cost calculation) are shown in Table 1. HIV/HCV co-infected patients were 26%, females were 59%, the most frequent infecting genotype was 1b, and median age was 47 years (interquartile range - IQR 17).

**Table 1:** Patient characteristics and treatment response (actual response to Peg-interferon + ribavirin or expected response after addition of directly acting antivirals)

	Characteristics	Catanzaro (HIV negative)		Padua (HIV positive)		Total	
		n = 77	(74%)	n = 27	(26%)	n = 104	(100%)
***Qualitative variables***	**Gender:**						
	Males (%)	37	(48)	6	(22)	43	(41)
	Females (%)	40	(52)	21	(78)	61	(59)
	
	**Genotype:**						
	1b (%)	60	(78)	16	(59)	76	(73)
	1a (%)	11	(14)	10	(37)	21	(20)
	1 (%)	6	(8)	1	(4)	7	(7)
	
	**RVR with PEG-IFN/RBV:**						
	nonSVR RVR (%)	2	(3)	0	(0)	2	(2)
	SVR RVR (%)	5	(6)	2	(8)	7	(7)
	SVR nonRVR (%)	41	(53)	9	(33)	50	(48)
	nonSVR nonRVR (%)	39	(38)	16	(59)	45	(43)
	
	**Response to PEG-INF/RBV (actual):**						
	SVR (%)	46	(60)	11	(41)	57	(55)
	Relapsers (%)	12	(15)	4	(15)	16	(15)
	Partials (%)	2	(3)	4	(15)	6	(6)
	Nulls (%)	17	(22)	8	(29)	25	(24)
	
	**Response to PEG-INF/RBV + DAA in naïves (estimated):**						
	SVR (%)	58	(75)	20	(75)	78	(75)
	Non response (%)	19	(25)	7	(25)	26	(25)
	
	**Response to PEG-IFN/RBV in naïves *plus *PEG-IFN/RBV + DAA in non responders (estimated):**						
	SVR (%)	62	(81)	19	(70)	81	(78)
	Non response (%)	15	(19)	8	(30)	23	(22)

***Quantitative variables***	**Age (at PEG-IFN/RBV start):**						
	Median	50		45		47	
	Range	20-70		36-60		20-70	
	IQR	23		7		17	
	Standard Deviation	14		5		12	
	
	**Duration of PEG-IFN/RBV treatment in weeks (actual):**						
	Mean	44		40		42	

**Figure 1:**
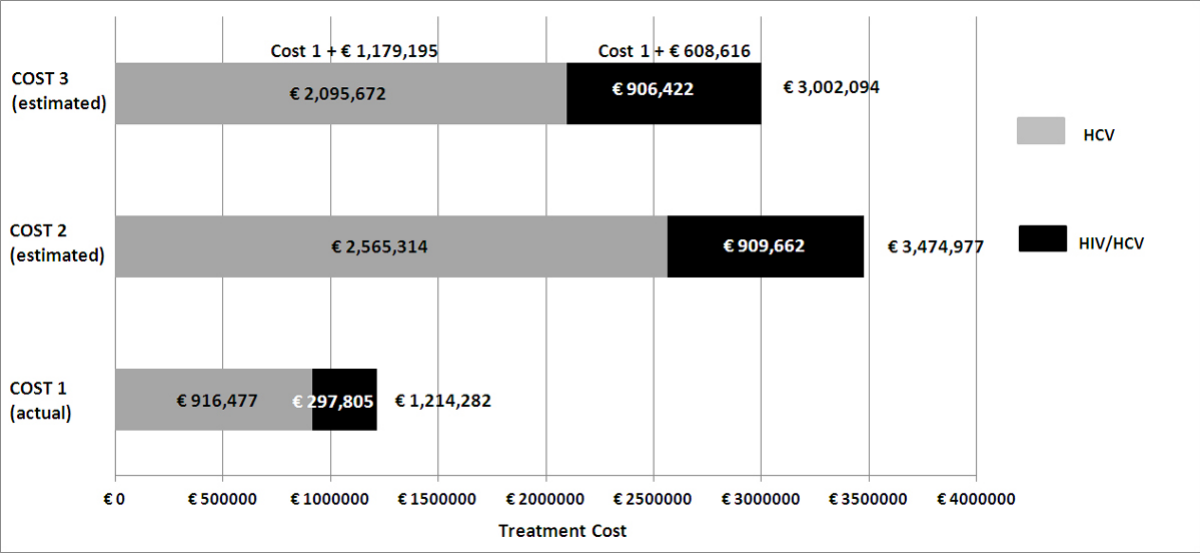
**Treatment costs (actual and estimated)**. NOTE: Cost 1 represents the actual cost incurred for treating the 104 patients under study with Peg-interferon and ribavirin while naïve. Cost 2 represents the estimated cost of a lead-in strategy with PEG-IFN/RBV for 4 weeks, followed by a directly acting antiviral for 12 or 36 weeks in the same patients while naïve. Cost 3 represents the estimated cost of PEG-IFN/RBV in naïve patients (Cost 1) *plus *PEG-IFN/RBV with addition of a DAA in our patients who did not achieve a SVR.

**Figure 2:**
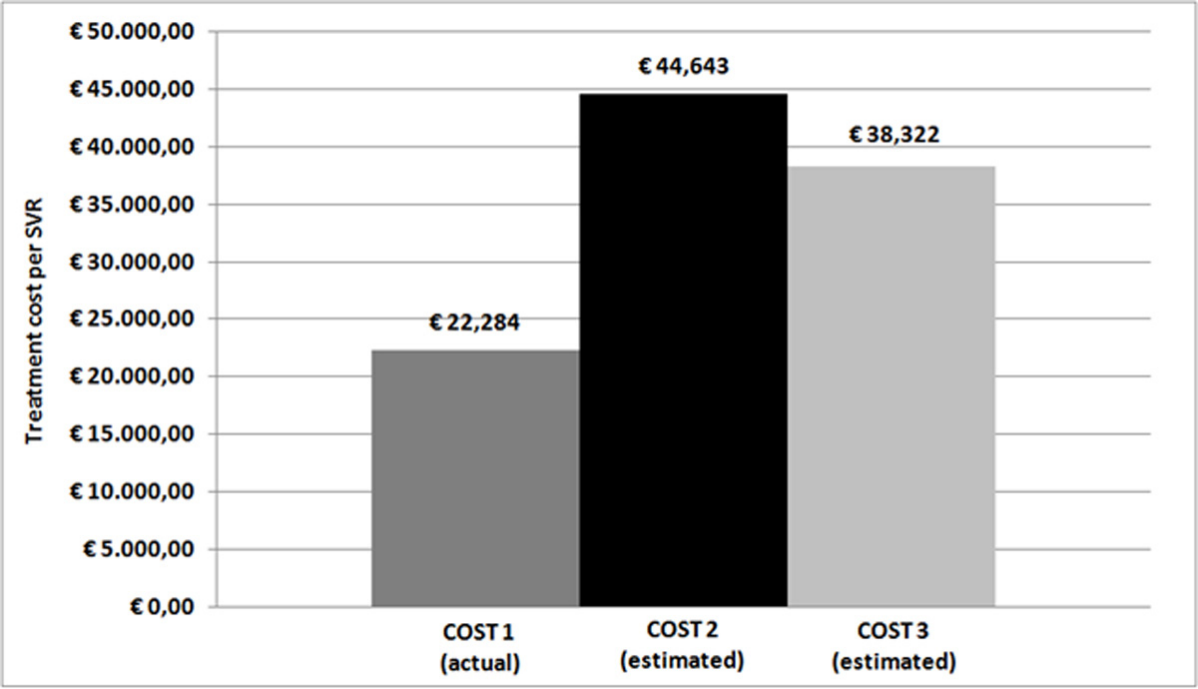
**Treatment cost per SVR (total costs were divided by the corresponding absolute numbers of SVR achieved or expected)**. NOTE: Cost 1 represents the actual cost incurred for treating the 104 patients under study with Peg-interferon and ribavirin while naïve. Cost 2 represents the estimated cost of a lead-in strategy with PEG-IFN/RBV for 4 weeks, followed by a directly acting antiviral for 12 or 36 weeks in the same patients while naïve. Cost 3 represents the estimated cost of PEG-IFN/RBV in naïve patients (Cost 1) *plus *PEG-IFN/RBV with addition of a DAA in our patients who did not achieve a SVR.

Treatment with PEG-IFN/RBV was completed by 89 of 104 patients (85%). In other patients, interruption of treatment was due to decision of patient (2%), drug related side effects (9%) and viral failure (4%). Rate of SVR was 55% overall (22% males, 33% females), while it was 60% among HCV mono-infected patients and 41% among HIV/HCV co-infected ones. Virological relapse was observed in 15% patients in both HCV mono-infected and HIV/HCV co-infected groups. Patients had a partial response in 6% of cases (15% among HIV/HCV co-infected and 3% among HCV mono-infected patients). Null responders were 24% (29% HIV/HCV co-infected and 22% HCV mono-infected patients). A RVR was reached in 9% patients, 48% reached SVR without RVR and 43% did not achieve either response. As shown in Figure 1, overall drug cost (cost 1) was €1,214,283 (€916,477 for treating HCV mono-infected patients; €297,805 for HIV/HCV co-infected ones), and cost per SVR achieved was €22,284 (Figure 2).

Patients who achieved a RVR would not need a DAA, but would only be treated with further 44 weeks of PEG-IFN/RBV combination. The remaining 95 patients would be eligible for DAA treatment. Among these patients, according to the results of the ADVANCE trial, 55 would receive only 12 weeks of PEG-IFN/RBV afterwards. The last 40 patients would need further 12 weeks of PEG-IFN/RBV and DAA followed by 36 weeks of PEG-IFN/RBV (according to 42% rate of detectable but ≤1,000 IU/ml HCV-RNA at weeks 4 or 12 of triple therapy) [9]. Using these estimates of virological response and length of treatment, overall cost 2 was estimated to be €3,474,977 (see Figure 1). Corresponding cost per SVR achieved resulted to be €44,643 (Figure 2).

Estimation of cost 3 was obtained by adding to cost 1 the cost for re-treatment with a DAA of the 47 patients who did not achieve a SVR with PEG-IFN/RBV. Total cost 3 would be € 3,002,094 (Figure 1). Cost for retreatment of patients would be €1,179,195 for HCV mono-infected patients and 608,617 for HIV/HCV co-infected patients. Thus, total incremental cost (cost 3 *minus *cost 1) would be €1,787,812. The average cost per SVR achieved would be €38,322 (Figure 2).

## Discussion

This study estimated costs needed for treating with a DAA containing regimen patients either experienced or naive for PEG-IFN/RBV. Moreover, we projected future costs against actual costs already incurred for treating the naïve patients with only PEG-IFN/RBV. Lastly, we compared costs for each SVR achieved. Our results will provide local health authorities with an estimate of resources needed to treat our patients with the currently available DAAs. Clearly, the study was not aimed at assessing cost-effectiveness. Further investigations using either Markov model or cost-effectiveness analysis calculations are therefore needed.

Patients who underwent treatment with PEG-IFN/RBV reached a SVR in 55% cases overall, with a difference between HCV mono-infected patients (60%) and HIV/HCV co-infected ones (41%). In HCV mono-infected patients, the rate of SVR in our study was even better that in two US registration trials, showing SVR rates of 54-56% [12,13]. However, according to registration trials, the rate of SVR after triple therapy is around 80% [9,10]. Therefore, our results support the benefit of triple therapy over dual therapy, which should be abandoned for treatment of patients infected by genotype-1 HCV. Indeed, first generation DAAs showed to be cost-effective either in naïves or IFN- experienced patients [11,14,15]. Moreover, second generation DAAs, such as Sofosbuvir, were proved to be cost effective in naive patients [16].

PEG-IFN/RBV has represented a scarcely effective therapy in HIV/HCV co-infected patients with genotype 1, as recorded in our study (41% SVR among HIV/HCV co-infected patients). These low SVR rates impacted on total drug expense. Indeed, although HIV+ patients represented only 26% of our population they would weigh for 34% of the entire sum needed for retreating experienced patients.

Because progression towards end stage liver disease (cirrhosis, hepatocellular carcinoma and death) in HIV/HCV co-infection is faster than HCV mono-infection, co-infected patients with significant liver fibrosis are urged to be treated for HCV as soon as possible [17]. During the 21^st ^Conference on Retroviruses and Opportunistic Infection (CROI), two trials of the French National Agency for Research on AIDS (ANRS) showed high effectiveness of either TPR or BOC based regimens among experienced HIV/HCV co-infected patients (79.7% SVR after 24 weeks from end of therapy with TPR, 53% SVR after 12 weeks from end of therapy with BOC). Rates of discontinuation due to adverse events (mostly haematological) were comprised between 10 and 20% [18,19]. Also, two real-life studies showed overall SVR rates of 64% after 12 weeks from the end of therapy with BOC or TPR [20,21]. At the same time, an interferon free trial highlighted response rates close to 100% at week 12 of therapy with sofosbuvir plus ledipasvir [22]. However, in settings where safer and more effective second generation DAAs are far from being available, TPR and BOC could offer reasonable chances to reach SVR, even if they are frequently affected by severe drug toxicity.

Although the benefits of a DAA containing therapy are well demonstrated (both for an increase in SVR and for cost-effectiveness in terms of years of life gained adjusting for quality of life), we found the cost for triple therapy including DAA would be much greater (a threefold increase) than for PEG-IFN/RBV. Furthermore, the cost for treating our patients who did not obtain a SVR after PEG-IFN/RBV with DAA will be slightly greater than the amount spent to treat these patients for the first time (€1,787,812 versus €1,214,283). Lastly, mean cost per SVR was two times greater for DAA than for PEG-IFN/RBV treatment. Therefore, our findings demonstrate that DAA will substantially impact the economical budget of the national health system; information which will be useful for planning future resource allocation. Estimations are expected to increase further if costs for monitoring and treatment of adverse effects would be considered. Although it is possible that these costs will decrease with newer regimens sparing PEG-IFN, the above considerations suggest the opportunity to decrease costs for therapies to improve sustainability. *Cammà et al*. [11] found that the application of a lead-in period with PEG-IFN/RBV would improve cost-effectiveness because DAA are prescribed only to patients who did not achieve a RVR measured at week 4. Furthermore, *Marcellin et al*. [23] demonstrated that patients with detectable HCV-RNA levels at week 4 but with a reduction of HCV-RNA by 3 log_10 _from baseline reached SVR in a high percentage of cases (61%). In the present study, we found that RVR rate in patients treated with PEG-IFN/RBV was low (9%), but several patients achieved SVR anyway (48%). This may be a reason why the estimated cost for DAA in patients who completed PEG-IFN/RBV without a SVR appeared to be smaller than the cost estimated for treating only patients who did not achieve a RVR with triple therapy, an approach that emulates the lead-in strategy. Therefore, we can argue that a prolongation of initial PEG-IFN/RBV could further improve cost-effectiveness in the context of a lead-in strategy. This may require further investigation.

Different subtypes of genotype 1 exhibit variable response to double and triple therapy. It has been proven that genotype 1a has a higher genetic barrier against emergence of HCV resistance to TPR and BOC than genotype 1b. Indeed, resistance to TPR and BOC in genotype 1a is caused by one single nucleotide substitution while at least two different nucleotide substitutions are needed in genotype 1b [24]. Also, a selection of a DAA molecule more specific or appropriate for the control of a particular genotype, will certainly contribute to improve cost-effectiveness and reduce the resources needed to treat our patients.

This study is affected by some limitations. First, TPR but not BOC was used for our estimations. Boceprevir is a bit less expensive than TPR but length of treatment is longer and number of pills is higher. For this reason, there is a tendency to prefer TPR as first choice. Further studies using BOC are required, however. Second, IL28B polymorphism was not assessed in our patients. IL28B has been shown to predict treatment response, so its consideration in conjunction with RVR would further improve cost-effectiveness [15]. Third, the cost impact of genotype 1 subtypes, as well as of other genotypes of HCV, was not evaluated in the present study, although it will be addressed in a further manuscript in preparation. Fourth, in our study, neither histological nor clinical conditions (such as co-morbidities and liver disease assessment) could be considered, so, it was not possible to infer appropriately about clinical challenges in treating our population with anti HCV drugs.

In conclusion, although cost effectiveness of TPR or BOC in combination with PEG-IFN/RBV has been established (when considering quality of life and prevention of long-term complications of HCV), from the perspective of the health care system, the cost for providing drugs may limit prescriptions; especially in the current economic crisis. So, we suggest that anti-HCV pipeline be urgently implemented towards the production of even more effective and cheaper drugs. While awaiting less expensive drugs, proper pharmaco-economical studies should be conducted to find more cost-effective schemes and indications for treatment in clinical practice.

## List of abbreviations

HCV, hepatitis C virus; PEG-IFN, pegylated interferon-α; RBV, ribavirin; DAA, directly acting antiviral; BOC, boceprevir; TPR, telaprevir; NHS, national health system; HIV, human immunodeficiency virus; RVR, rapid virological response; EMA, European medicines agency; HAART, highly active antiretroviral therapy; IL-28B, interleukin-28B.

## Competing interests

The authors declare that they have no competing interests.

## Authors' contributions

AS collected data, contributed to data analysis and to manuscript writing; CC, VP, VDM, FG, SDS, SGP, MB, MMF collected clinical data; NM carried out laboratory analysis and contributed to manuscript revision; MCL contributed to manuscript revision; GSB, AGL carried out laboratory analysis; EZ carried out laboratory analysis and contributed to manuscript revision; MCP, GM, AF contributed to manuscript revision; CT contributed to manuscript writing.

## Declarations

Publication of this supplement was partly supported by an unrestricted grant provided by Roche. The articles were independently prepared by the authors with no input from Roche. Roche were not involved in selecting the articles for the supplement. The pegylated IFN/pegIFN treatment mentioned in this article is produced by Roche.

This article has been published as part of *BMC Infectious Diseases *Volume 14 Supplement 5, 2014: Proceedings of the Third Workshop of the HCV Study Group in the Calabria Region: Results from the South Italian Network for Rational Guidelines and International Epidemiology (SINERGIE) Project. The full contents of the supplement are available online at http://www.biomedcentral.com/bmcinfectdis/supplements/14/S5.
